# Round the Hospitals

**Published:** 1920-04-17

**Authors:** 


### ROUND THE HOSPITALS.

BEST wishes for a record success to the Bazaar taking place at Claridge's Hotel on Thursday in this week in aid of St. Mary's Hospital for Women and Children at Plaistow. Princess Marie Louise is selling groceries, and the complete clearance of her stock at what may be called profiteers' prices is a foregone conclusion. The Duchess of Wellington has some beautiful old glass and antiques, well worth alone the visit of the connoisseur, and Lady Mary Cambridge, Katherine, Duchess of Westminster, the Dowager Countess of Leicester, and other notables are lending aid. The special purpose for which the money is being raised is the erection of a Nurses' Home. At present the

**ROUND THE HOSPITALS**—(*continued*).

nursing staff are housed not very comfortably outside the hospital, and have to cross a piece of waste land and a street before reaching the grounds of the institution. Strange as it may seem to the advocates of the living-out system, nothing puts a greater strain on the endurance of nurses than having to brave all kinds of weather at all times of the night and day when they pass to and fro between the wards and their own quarters. The continued growth and prosperity of St. Mary's Hospital will receive its fitting crown when the nurses are properly housed in the midst of this somewhat depressing area.

---

The new V.A.D. Club for present and former members of Voluntary Aid Detachments is achieving a most striking success from the outset. An admirable establishment, formerly an hotel, has been secured in Cavendish Square, and from forty to fifty bedrooms will be available at moderate rates. Already over 2,000 members have joined. The founders are Lady Amphill, Lady Oliver, Dame Rachel Crowdy, Lord Donoughmore, Lord Chilston, Sir Arthur Stanley and Mr. R. Holland Martin. The funds are being largely contributed by members who are taking up shares in the undertaking and forming a limited company. An interesting feature of the Club is that the staff will, if possible, consist entirely of V.A.D. workers, general service members, etc., and it is hoped to secure as high a standard of cooking and general smartness as that which distinguished the best V.A.D. hospitals. A cafetière buffet will be provided in addition to the dining-room restaurant, and here V.A.D. workers who are not members of the Club can have meals if they are wearing uniform or can produce their certificate. Quite apart from its individual advantages to members, the Club is likely to be of public service as forming a centre for reunions of V.A.D. workers and promoting camaraderie among them.

---

The *Daily Telegraph* Fund now amounts to 232,211 shillings, and the sums recently received fully prove the wise and generous policy of the proprietors in continuing their labours on behalf of the nursing profession, for nurses themselves and others associated with their interests are still working hard on their behalf, and news of the Fund is constantly filtering through to fresh quarters. We are persuaded that there is much more to come before the fount of liberality for this great purpose runs dry.

---

Considerable interest has been caused institution circles by a letter of Sir Henry Morris to *The Times* protesting against official estimates as to increase of cost of living to-day as compared with pre-war years. He gave a summary of his own expenses for food, drinks, laundry, coals, etc., and showed that comparing 1916 with 1919 the increase in the latter year was only 23.3 per cent., while in 1916 the increase over 1913 was only 1.05 per cent. Other persons bear out his contention that the increase of 50 or 60 per cent. demanded in wages or salaries to meet the extra cost of living is not justified by the facts. The headmaster of a grammar school estimates the difference at 25 per cent. in the upkeep of his establishment. We would point out that the demands for increased salaries are not exclusively based on the higher cost of living. In the case of nurses the condition of the labour market has even more to do with their just demands for extra pay than the higher cost of food, which hardly touches them individually at all, if the case of district nurses be excepted. In institutions, as in private families, the changing value of foodstuffs has proved the housekeeper's test. But no estimates which include the rationed years are of much use. The pull towards economy was then irresistible.

---

Lady Leigh, whose name occurred as recipient of the title of Commander of the Order of the British Empire in the recent list, is one of the women who made a thorough study of hospital equipment during the war, and may be considered an expert in this difficult branch of knowledge. Her husband, Sir John Leigh, established two hospitals for the wounded, which were equipped, and for some time managed under the personal direction of Lady Leigh, one at Altrincham for eighty wounded officers, and another, the John Leigh Memorial Hospital, at Brooklands. The Altrincham hospital has now been handed over to the Ministry of Pensions as a training centre for officers suffering from shell-shock, while Brooklands is still in use. We are glad to learn that Lady Leigh is a member of the Board of Management of Llandudno Hospital, and trust that her past experience will bear good fruit.

---

National Health Week, which is to be celebrated at the beginning of May, will be more than usually significant in its appeal this year to the public. Undoubtedly the right ideal is for the spirit of health to be unconscious. The healthiest children are those who hear nothing about "constitutions," and the sturdiest members of society may be seen taking with impunity all the liberties they ought not to take with their physical powers. But this condition of subconscious health has long been lost to the community at large, and can only be regained by a prolonged and united effort. People must be taught, line upon line, precept upon precept, the lessons which lead to security and independence in matters relating to their bodies. "Self-help" is to be the motto for this year's Health Week, for authority, as represented by medical officers of health, has done almost as much as it can in the way of compulsion. The King and Queen are patrons of the National Health Week movement, and the chairman is the Lord Mayor of London. The workers who can best set forward its aims are the nurses, and, in private practice as well as in district work, every nurse

ROUND THE HOSPITALS—(*continued*).

should set herself as a task during the coming week to choose out for illustration one or two vital matters of self-help concerning the well-being of her patients, and make sure that her precepts are understood.

---

The Board of Management at the Blackburn and East Lancashire Royal Infirmary have made further additions to the salaries of the nursing staff as from April 5, 1920. The new scale of salaries marks a great advance on the revision made a year and a-half ago. Probationers are now to receive £20, £25, £30, in lieu of £16, £22, £25, in respective years. Staff nurses (after third year) are to receive £55, £60, in lieu of £40. Ward sisters, whose previous salary ranged from £45 to £55, are to receive £65, £70, £75, £80; theatre and night sisters £80, £85; assistant matron £100. The matron's salary rises from £150 to £200. We are confident that the managers will reap a rich reward from this liberal policy, and that the extra income required to carry it into effect will not be grudged by the subscribers.

---

Preparations are already on foot for amending the machinery for the census next year, which is to be taken on the first Sunday in April. Dr. Addison is preparing the necessary measures for obtaining Parliamentary sanction for the returns in Great Britain and Ireland, and we trust he will take steps to discriminate as regards occupation between trained nurses and nursery-maids. A half-hearted attempt was made ten years ago to get the awkward and misleading nomenclature amended, but nothing much came of it and we are still without any official computation of the number of women who are qualified satisfactorily or otherwise to act as sick-room nurses. It is a matter which lies entirely within the province of the Health Minister, and we cannot believe there is any insuperable difficulty in getting a workable definition of the word "nurse," on which to base statistics.

---

Recently a Committee of the Camberwell Board of Guardians was asked to go into the matter of training nurses in midwifery at one of the institutions. The committee now reports in favour of such a scheme, suggesting that in future, after the passing of the final examination by probationer-nurses, the Guardians should select four, each year, who should be permitted to sign on for a further year for the purpose of being trained in midwifery. The scheme proposed is that each of the four nurses should serve three months in the Constance Road institution (receiving the salary of an infirmary staff-nurse) in order to obtain the practical experience (twenty cases) required by the regulations of the C.M.B., and the other nine months in the infirmary as staff nurses, at the same time receiving from the medical superintendent the qualifying lectures.

A motion to alter the name of the Camberwell Infirmary to "Camberwell Municipal Hospital" was lost at a recent meeting of the Guardians. It would be regrettable if the good old word "Infirmary" had so severe a slur put upon it. It is true that by parsimony and what may be called strong-heartedness the Guardians of the Poor in some localities have caused the word to stink in the nostrils of the people for whose comfort it was devised. But these ill associations can be lived down, and none are doing better service in restoring confidence in the "Infirmary" than the Camberwell Guardians themselves. We note with satisfaction that since the 48-hour week for nurses has been in force at this institution the old, wasteful practice of saving up meals for officers off duty, much as cold food used to be put aside for recalcitrant children in days gone by, has been abandoned and a shocking waste of food prevented. Allowances have now been agreed upon in lieu of board or partial board to officers of the institution during absence on leave, at the rate of 2s. for a full day or night, and 1s. 4d. for dinner and tea. It is not excessive, but a step in the right direction. A medal is, for the future, to be awarded to the most efficient all-round nurse of the year.

---

The Invalid Children's Aid Association, which will hold its annual meeting at 3 p.m. on April 27 at Londonderry House, Park Lane, is among the philanthropic agencies in the Metropolis which can least be suffered to go lacking in support. The principle of finding a friend among the general public for each of the ailing children to whom it ministers is admirable both in its effects on the child and on the friend. The institution of a Golden Book has been devised to interest people in individual children. All sums of money subscribed by sympathisers are devoted to the welfare of some invalid child, a report of whose progress is sent from time to time. The annual meeting of the Society is to be a great rally on its behalf. The American Ambassador will be in the chair, and other speakers will be the Bishop of London, Miss Irene Vanbrugh, Gerald du Maurier, and Major Beith (Ian Hay). Admission is by ticket, to be obtained from the Secretary, 69 Denison House, 296 Vauxhall Bridge Road, S.W. 1.

---

It is always satisfactory when efforts begun in war-time become permanent additions to the philanthropic resources of the country. The Woolwich Nursery for Children of Munition Workers has kept open until lately, when it was handed over by its organiser, Lady Henry, to the London County Council as a gift in memory of her only son, Lieutenant Cyril Henry, who fell early in the war. It is to be used as a children's hospital for minor operations, and the staff has been transferred to a new crèche which Lady Henry and Mrs. Mitchison are organising in Kensington. The nursery at Woolwich, open night and day during its term of occupation, boasted nearly 52,000

**ROUND THE HOSPITALS**—(*continued*).

attendances. The Duchess of Albany, hearing that it was about to close down, paid it a visit recently and spent several hours among the children.

A very sad case came before the Coroner at West Byfleet on April 8 in the suicide of Nurse Ada Minnie Spreadbury, aged thirty-three, health visitor in the district. Evidence was given to show that she had had some training as a nurse and was a midwife. She had been employed by the Surrey County Council since November 1917, but latterly had suffered much from depression, and had threatened either to go into a convent or take her own life. She was in great mental distress from the delusion that her friends and relatives were against her, and that the people in the district were suspicious of her. She burnt her clothes and other possessions, and was discovered lying unconscious in bed, with her face much discoloured and an empty bottle which had contained chlorodyne by her side. The medical evidence showed the cause of death to be narcotic poisoning. Mr. Henry Jones, the county medical officer of health, spoke highly of Miss Spreadbury as a most conscientious and painstaking health visitor. He had formed an opinion lately, however, that she was suffering from incipient insanity, and had warned the relatives to that effect. The verdict was "Suicide while of unsound mind."

The Tynemouth Board of Guardians are much resenting the refusal of the Board of Education to recognise their fine Poor-law Infirmary for the purpose of their regulations as to the allocation of grants, as a midwifery training-school. No objections are made to the institution, which is exceptionally well equipped and managed in every particular. It is the fact that it is conducted by Guardians of the Poor which is held to disqualify it for the receipt of the midwifery grants made to other institutions, and in this decision the Ministry of Health concurs. The Chairman of the Board, in a stirring speech, has protested against the "gratuitous insult," and lamented that the presense of lady Guardians prevented him from expressing himself "in language suitable to the occasion." A sub-committee was appointed to negotiate with the Board of Education and the Ministry of Health.

The Lewes Workhouse Infirmary appears to be in a very unsatisfactory condition as regards its nursing arrangements, to judge from a recent discussion of the Guardians. Dr. Fuller and Mr. G. R. Snowdon, Ministry of Health Inspectors, attended a meeting of the Workhouse and General Purposes Committee and made recommendations concerning the number of nurses and their salaries, dietary, and hours of work, to which the Guardians demur. At present there is but one trained nurse besides the head nurse. Dr. Fuller urges the appointment of an additional trained nurse, to supplement the three untrained nurses "with experience." He considers the salaries are inadequate and ought to be raised; that two more nurses' bedrooms are required; that a servant should be engaged for the nurses' home, and that the infirmary should be provided with an efficient hot-water supply, the boiler having been allowed to go out of repair. These recommendations appear to us to be moderation itself, and as the Guardians for lack of attractions for their staff are now often reduced to engaging nurses at £2 2s. a week and pay out a good deal in advertisements, we trust wiser counsels will prevail and a sound policy be shortly inaugurated.

The immediate future of probationers after the completion of their course is a subject of perennial interest. For that reason we are grateful to Miss Babtie, Matron of St. George's Hospital, for the clear synopsis of the training-school changes contributed to the annual report of the hospital. Out of the twenty-two staff nurses who have resigned, only one has done so for reasons of health. The remainder, on completion of their service, were accounted for as follows: Royal Air Force, 1; sanatorium in Switzerland, 1; nursing homes, 6; private nursing, 2; sister and temporary sister at St. George's, 2; sister at another hospital, 1; midwifery training, 1; return to home life, 5; married, 2. The small proportion of nurses who have taken up private nursing is a testimony to the increased advantages now offered in institutions which absorbed altogether ten out of the available nineteen nurses. At present prices the charms of living out, even between cases, have suffered eclipse, while every effort is being made to render nurses' homes truly attractive.

Australia boasts the pioneer nursing sister to become an air-pilot. Her name is Sister Hilda Hope McMaugh, and on her return to Melbourne recently she awoke to find herself famous. She was a member of the Australian Imperial Force and served in Egypt during the war. Later she came to England and qualified for an air-pilot's certificate at the Northolt Aerodrome, where she did a good deal of flying, looped the loop, and ascended to a height of 12,000 feet. Her instructor was Captain Sykes, O.B.E. Her interviewer elicited the fact that she had had one slight accident in descending to the aerodrome in the dark when a horse and cart were crossing the ground, but though she alighted on the cart she escaped injury and did not even damage the machine.

The continued shortage of probationers in the manufacturing districts makes it most difficult, even in the best-equipped hospitals, to keep the staff up to strength. In the annual report of the General Hospital, Birmingham, just issued, we note that the number of the nurses has been much below the authorised number throughout the year, and this has thrown a great strain upon the existing nursing staff. The Board expresses gratitude for the way in which the nurses have met the extra work thrown upon them.

